# Exploring the repeatability of curved gait during an instrumented figure of 8 walk test, a new methodology

**DOI:** 10.1038/s41598-026-57567-2

**Published:** 2026-06-18

**Authors:** Nicolas Prieur-Blanc, Adrien Mangini, Samuel Trama, Dorian Giraud, Denis Bertin, Laurent Bensoussan, Arnaud Hays

**Affiliations:** 1 Aix-Marseille Univ, CNRS, HIPE Human-Lab (UAR 202324378), Campus Timone 27 boulevard Jean Moulin, 13005 Marseille, France; 2 Physical and Rehabilitation Medicine Department, APHM, South Marseille University Hospital, Marseille, France; 3 Aix-Marseille Univ, CNRS, ISM (UMR 7287), Marseille, France; 4 Polytech Marseille, Aix-Marseille Univ, CNRS, IUSTI, Marseille, France; 5 Aix-Marseille Univ, CNRS, INT (UMR 7289), Marseille, France; 6 UGECAM Institut Universitaire de Réadaptation de Valmante Sud, Marseille, France

**Keywords:** F8W, Gait, Turning, Barefoot, Shoes, Walking speed, Walking test, Engineering, Health care, Medical research

## Abstract

There is currently no consensus on how to assess curved gait. One of the challenges is to define when a turn begins and ends. Two fundamental characteristics of a turn are the variation in gait speed and the vertical rotational rate of the pelvis. Our study aimed to assess the repeatability of an instrumented figure of eight walk test (F8Wi) by evaluating right and left turns over 3 sessions. Fourteen healthy participants were included. Participants walked barefoot and with shoes, both at comfortable and fast speed. Several points of interest were identified: the start of speed deceleration (A), the start of body rotation (B), the peak angular acceleration (C), the end of body rotation (D), the end of speed acceleration (E). The dependent variables were time intervals between AB, AC, AD, AE, BD, and gait parameters measured during the turn defined by time between A and E: mean sum load from the lumbar inertial measurement unit, mean sum load from the lumbar inertial measurement unit normalised by AE time and mean of the right knee maximum flexion of each step during the turn. The coefficient of variation (CV), standard error of measurement (SEM), minimum detectable change (MDC_95%_), and 3D Bland‒Altman plot assessed repeatability. For all conditions, the CVs of time between AE, AD, and BD were less than 10%, whereas for times at AB, AC, AD, BD, the CVs were between 10% and 20%. The SEMs remained low for all variables and conditions, and the MDC95% was greater than the standard deviation. For the gait parameters, CVs were less than 10%, the SEMs remained low, and the MDC_95%_ was greater than the standard deviation. The protocol employed a combination of knee electrogoniometers, an IMU and a Doppler radar to evaluate curved walking during an F8Wi modified. Curved walking repeatable over time and under different gait conditions. This repeatability study shows that a more accessible setup could open the door to assessing curved walking.

**Supplementary Information:**

The online version contains supplementary material available at https://doi.org/10.1038/s41598-026-57567-2.

## Introduction

Humans do not only walk in straight lines to explore and interact with their environment. Turning and curved walking are important components of daily activity, accounting for 35–45% of the walking time^1^. Evaluating gait during turning is challenging due to its variability. For example, responses to a demand to change direction may vary depending on when the cue is given, introducing the concept of turn strategies (e.g., spin and step turns)^2^. A spin turn involves spinning the body around the inner leg of the turn, while a step turn consists in employing a sequence of steps to perform a turn. For healthy individuals, straight walking is considered an almost automatic action, with no cortical relay^3^. However, it has been suggested that turning is not an automatic process and instead requires cognitive processing^3^. Indeed, oxygenation of the prefrontal cerebral lobe changes during curvilinear walking compared to walking in a straight line when a dual task is performed^4^. These observations underscore the benefits of studying curved walking in relation to human motor physiology and its links with cognition^5^.

Curved walking is included in certain routine clinical assessments, such as the Timed Up and Go (TUG) test^6^. The TUG involves a series of activities: standing up from a chair, walking in a straight line, turning around, and sitting back down. However, its results only cover the total time it takes to perform all these tasks, as well as some descriptive elements (e.g. stability, accuracy) that occur during the test. Another limitation is that a single TUG trial is often insufficient for pathological cases. For example, it is stated that obtaining the TUG time after a hip fracture requires three attempts, and the average of these is used^7^. Furthermore, only one direction of turn is tested during a TUG test. Son and Park, 2019^8^ demonstrate that the time taken to complete the TUG after hemiplegia depends on how the turn is taken. Moreover, people with hemiplegia after a stroke won’t spontaneously turn in each direction depending on the laterality of the hemiplegia during a TUG^9^. To resolve this issue, it is advisable to readminister the test and examine each side^8^. To quantify TUG, an instrumented TUG (TUGi) has been created, the TUGi uses an inertial central unit to measure the time it takes to turn during the test^10,11^. The 180° turn analysis of TUGi by IMU appears to be repeatable over time in both healthy people and those with Parkinson’s disease^12^.

However, the study by Viteckova et al.^12^ does not mention the laterality of the turn. To counteract the one-sidedness of the shift, the figure of 8 walk test (F8W) can be used, as it focuses on gait and explores both sides of the turn. The F8W, which is 1.5 m long, has been validated in various populations of people suffering from Parkinson’s disease, multiple sclerosis, stroke, leg amputation, arthritis, or geriatric conditions^13–15^ and is repeatable in some conditions (e.g. after knee replacement, multiple sclerosis, stroke)^16–18^. The F8W is also used in fundamental research^4^. Moreover, the continuity of the traditional eight shapes, makes it possible to link different eight shapes together while avoiding the start and end points, which can modify gait parameters^19–21^. The F8W was initially conceived for routine use in medical consultations where space is limited and only one 8-shape was performed. Speed was assessed using a stopwatch to time how long it took a participant to complete the F8W, while amplitude was calculated by dividing the total distance by the number of strides. Descriptive elements, such as smoothness and stability, were also noted, but not quantified using measurement devices^13^. It has to be noted that the F8W is performed at 1.5 m which is insufficient to achieve a regular walking speed^22,23^. Acceleration and deceleration phases of straight walking occur between the two turns of the F8W, modifying gait parameters^19^. A short distance may therefore experimentally affect walking speed, just as the positioning of start and stop points can artificially influence it^20,24^. To minimise variations in gait patterns, a modified F8W was proposed, featuring two straight sections of 3.7 m each and two curved sections with a radius of 1 m; only one 8 shape is permitted during each attempt due to this new form of 8^25^. Zanca et al.^25^, however, suggest taking into account the averages of the entire test, with no difference between straight and curved gait. In total, this modified F8W allows participants to maintain a sufficient distance during a figure of 8 walking test, enabling them to accelerate and decelerate. The traditional F8W makes it possible to link the different 8 shapes, avoiding start and end points. A potential upgrade to the F8W would be to conduct a more precise evaluation of straight and curved phases by segmenting the turn analysis and measuring dynamic elements, while avoiding static landmarks^26^.

Pelvic rotation in the horizontal plane is a key factor in detecting turns and has been quantified using marker-based 3D motion capture systems^27,28^. However, motion capture with markers has several drawbacks, including its cost, limited availability, as well as some uncomfortable situations related to nudity condition^29^. In addition, markerless systems still require pathology research^29^. El-Gohary et al.^30^ and Mancini et al.^31,32^ used an inertial measurement unit (IMU) placed at the 5th lumbar vertebra to detect the vertical rotational rate to determine the start and end of each turn. The TUGi uses antero-posterior acceleration to segment turns, as well as exploring vertical rotational rates^10,11^. Nevertheless, using the vertical rotational rate to detect turns via IMU may overlook differences in motion capture that arise from different turning strategies, such as the variation in kinematics between step and spin turns of the ankle, knee and hip joints^2,30^. Shin et al.^33^ reported that a turn has speed variations, starting with speed loss and ending with a return to average linear speed. Variations in walking speed are reproducible in a straight line using Doppler radar, but it has never been demonstrated in curved walks^20^.

Gait is rich in terms of different ways of walking^34^. Gait parameters can vary depending on the speed imposed during walking^35^. The turn is also affected, as a slower gait speed can modify the gait parameters and their variability during a turn^28^. The gait can be influenced by environmental conditions such as wearing shoes or going barefoot. Indeed, D’Août et al.^36^ and Franklin et al.^37^ compared gait parameters and reported differences between native barefoot walkers and people wearing shoes. Differences in gait parameters in the same person between walking barefoot or with shoes during straight walking were reported by Hollander et al.^38^. Variations in gait parameters due to shoes also need to be considered during clinical evaluation, Roman de Mettelinge et al.^39^ describes the variability in gait parameters for older women during gait evaluation due to walking barefoot. These elements, presented in a straight line, may occur during a turn. In clinical gait assessment, it is necessary to assess people walking barefoot to investigate clinical signs of pathology^40^. Barefoot walking is repeatable over time in a straight line, as presented by Soulard et al.^41^. Hence, gait assessment must consider different walking conditions, such as walking barefoot or in shoes, and at different speeds, to be holistic and meet gait analysis standards^40^.

To ensure internal consistency, it would be interesting to explore the gait parameters using this new segmentation methodology and establish the repeatability of gait parameters during the turn. Our strategy uses different devices. Electrogoniometers measure kinematic data and avoid the need for motion capture^42^. The angle of knee flexion during a turn has already been shown to vary compared to straight gait^43,44^, but the repeatability of knee flexion during a turn has not been described. Furthermore, Hase et al.^2^ described joint flexion of the hip and knee, and presented differences between step and spin turns. These elements need to be taken into account when studying turning strategies. As we use an IMU to detect the rate of vertical pelvic rotation, it would be interesting to evaluate the workload of turning to the right and left over three sessions and observe whether this load is repeatable^45^. The workload is a feature clinicians can easily assess when managing patients^45^. It is also used to monitor Achilles tendinopathy and presents interest in terms of clinical management^46^. It would be interesting to monitor the repeatability and stability of this measurement during turning activity.

We proposed creating an instrumented F8W (F8Wi), which would link the different walk shapes, increase the distance between the turns and allow testing different gait conditions. At this point, we can identify two different approaches to analysing turn segmentations: one focusing on vertical rotational rate and the other on speed variation. It would be interesting to combine these two approaches using a new F8Wi methodology involving a speed Doppler radar and an IMU to quantify the segmentation and avoid the length limitations of the traditional figure of 8. The stability of this new methodology must be assessed using test-retest repeatability to ensure both performance reliability and measurement consistency^47^.

The aim of this study was to analyse the test-retest repeatability of a curved gait during an F8Wi, taking into account various speed and shoes conditions, with a particular focus on the different phases of the turn. We hypothesised that both the start and the end of the curved phase would be determined by the variation in linear velocity and vertical pelvic rotation rate during the F8Wi. These elements would be repeatable over time and for each gait condition when tested in healthy young adults. Our second hypothesis was that the following gait parameters during the curved phase would be repeatable over time and in each condition, as assessed with several tools (e.g., Doppler radar, IMU, and electrogoniometers) in healthy young adults: mean of the maximum knee flexion (MKF); mean sum load from the lumbar IMU (SLL).

## Methods

### Participants

The inclusion criteria were age between 18 and 40 years, no gait problems, ability to walk without limitations, and no reported lower limb or spinal events in the previous 6 months. The criterion for non-inclusion was the presence of a pathology that affected gait, such as a neurological or orthopaedic disorder that impaired walking over the past year. These elements were assessed using a questionnaire that asked the participants about their medical history. The questionnaire was administered by a medical doctor. Full ethical approval was received from the national committee CERSTAPS (reference: IRB00012476-2023-12-05-248). Written informed consent was obtained from all participants. The study was performed in accordance with the Declaration of Helsinki and met international standards in the exercise sciences^48,49^.

Fourteen participants were included (Table 1). All participants took part in three sessions. Ten had a right-handed dominant preference; eleven out of the fourteen participants demonstrated a right leg dominant preference (Table 1).

**Table 1:** Description (mean (standard deviation)) of the participants.

	Female	Male
Number (*n*)	6	8
Age (years)	24.3 (4.4)	24.5 (3.5)
Height (cm)	162.8 (3.0)	176.4 (9.3)
Mass (kg)	56.5 (12.0)	71.9 (12.6)
Body mass index (kg/m^2^)	21.3 (1.4)	23.1 (3.2)
Right-handed dominance (n)	4	6
Right leg dominance (n)	5	6
People practicing sport (n)	3	8
Wearing glasses (n)	3	1

### Data acquisition

This repeatability protocol involved each participant completing the F8Wi three times, with sessions scheduled on three separate days within the same week. During the first session, participants completed a questionnaire on their use of glasses, sports activities, and dominant hand and foot^50^. They were then measured and weighed using Tanita scales (MC980MA, Tanita Corp, Tokyo, Japan).

**Fig. 1:**
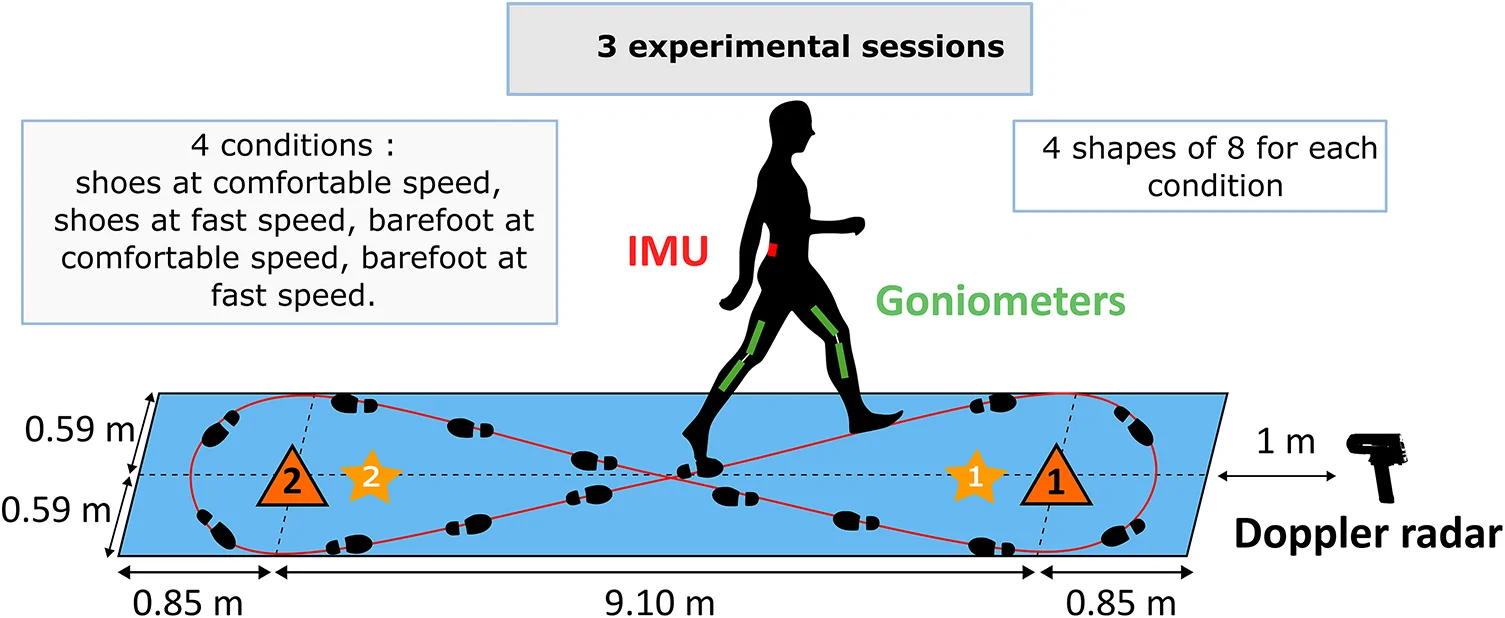
Schematic of the gait assessment installation during the F8Wi procedure. Triangles 1 and 2 correspond to cones 1 and 2. The star containing the number 1 represents the starting zone and the star containing the number 2 represents the end zone.

The experiments were performed in the same physiology laboratory (Fig. 1). The path length was 10.80 m, the distance between the cones was 9.10 m and the path width was 1.18 m. A speed Doppler radar (Stalker Pro II, Stalker Radar, US, 46.875 Hz) was positioned on a tripod at 1.85 m from cone 1 to measure the walking speed and variations in the straight linear phase of the F8Wi (Fig. 1)^51^. Two electrogoniometers (Biometrics Ltd., Gwent, UK, 1 kHz) were taped to the right and left knees^52,53^ to measure angle variation during walking and turning. One IMU was taped to the spine on the 4th lumbar vertebra (Cometa WaveTrack, MI, Italy, 2 kHz) to assess linear and angular acceleration (X-axis: antero-posterior; Y-axis: medio-lateral; Z-axis: vertical acceleration)^30^. Four force plates (Kistler 9667AA0906 (900 × 600 × 100 mm), Kistler, Switzerland, 2 kHz) at each turn were integrated below the tartan floor (X-axis: antero-posterior; Y-axis: medio-lateral; Z-axis: vertical forces). The eight force plates were coordinated as one. There were no differences in thickness or floor material. Force plates were delineated by some visual markings on the floor. Participants were instructed not to leave the zone during rotation. Analyses of the force plate data will be presented in a future publication.

The participants started at cone 1 and stood up for 3 s on the force plates to synchronise the different devices (Fig. 1). Then, participants were asked to make 4 shapes of 8 (Eight 180° turns implies four turns to the right and four turns to the left), each of which met one of the following randomly selected conditions: barefoot at comfortable speed (BC), with shoes at comfortable speed (SC), barefoot at fast speed (BF) or with shoes at fast speed (SF). For foot conditions, participants were asked to wear their usual shoes, with the exception of shoes with heels. For the fast speed condition, participants were instructed to walk at a speed that would allow them to catch their bus if they were late^54^. Once the 8th turn was executed, the participants were asked to go to cone 2 and stand up for 3 s in front of it to synchronise the different devices. Additionally, all the participants performed this procedure as a familiarisation before the first session. The order of the conditions was randomised in the first session of the participants, and the same order was maintained in session 2 and session 3.

### Data analyses

**Fig. 2:**
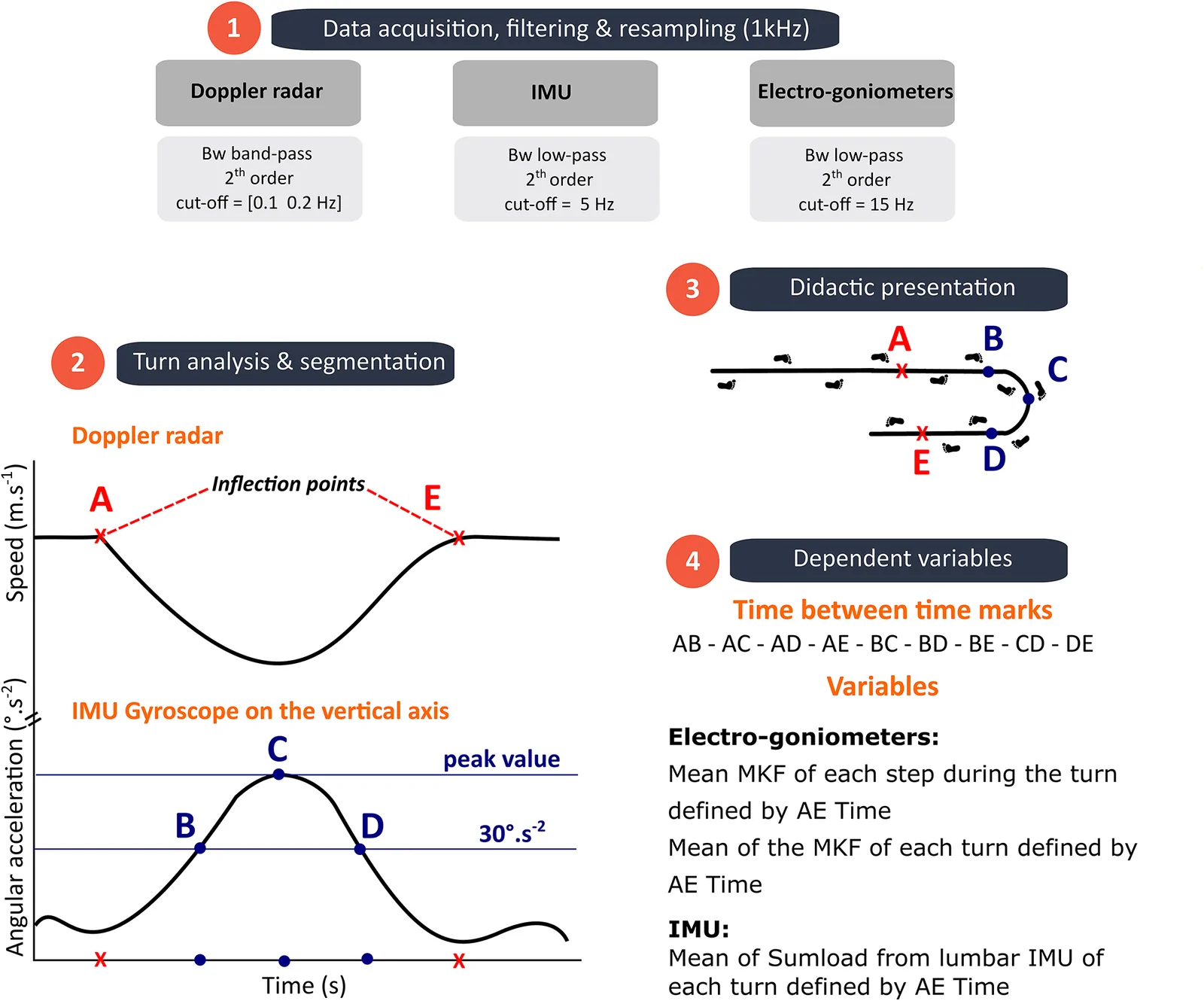
The following scheme illustrates the process of data acquisition and subsequent treatment. Bw, Butterworth; IMU, inertial measurement unit; MKF, maximum knee flexion; time mark A is identified as the start of the speed deceleration measured by the speed doppler radar, time Mark B corresponds to the IMU detecting an angular acceleration equal to or greater than 30° s^−2^ along the vertical axis, corresponding to the onset of axial rotation, time mark C is the peak angular velocity, at time mark D, the IMU detects a vertical angular acceleration equal to or less than 30° s^−2^ along the vertical axis, corresponding to the end of axial rotation, the average speed stops increasing at time mark E, measured by the speed doppler radar, corresponding to the end of the acceleration.

All data analyses were performed in MATLAB software (2023a, Matworks Inc., Novi, USA). The devices were synchronised by performing a stereotyped movement. Participants were instructed to perform a squat jump with a 90° knee flexion in a short period of time, with their hands on their trunk, and to jump and stand with their legs stretched out before the start of the F8Wi. At the beginning of the test, the take-off of a squat jump at cone 1 enabled synchronisation of goniometers, IMU (the peak acceleration along the vertical Z-axis of the IMU-accelerometer), and force plate (the peak force along the vertical Z-axis). The end of walking was marked by a standing position at cone 2 which enabled synchronisation of radar (the radar’s return to zero speed) and the last heel strike on the platform (as indicated by the final peak of force along the vertical axis). Subsequent manual verification was performed during data treatment to verify the quality of the signal on each piece of equipment and to verify the quality of synchronisation. The IMU and force plates data were resampled at a frequency of 1 kHz in accordance with electrogoniometers, keeping one point out of two. The data were filtered via a Butterworth filter. The cutoff frequency was determined by analysing the frequency spectrum of the signal (via fast Fourier transform) and selecting a frequency just above the range of meaningful signal content (Fig. 2, part 1). The start and end of the turning phase, i.e., deceleration, rotation, and acceleration, as well as the specific moments (e.g., the maximum angular velocity of the turn), were calculated as follows (Fig. 2, part 2):

- Start of the speed deceleration (point A): identified at the first inflection point of the Doppler radar speed curve^55^. This point marks the end of the straight gate phase.
- Start of the body’s rotation or onset of axial rotation (point B): identified with the IMU, detecting an angular acceleration equal to or greater than 30° s^−2^ along the vertical axis^30^.
- Peak angular velocity of the turn (point C): identified with the IMU along the vertical axis.
- End of the body’s rotation or offset of axial rotation (point D): identified with the IMU, detecting an angular acceleration equal to or less than 30° s^−2^ along the vertical axis^30^.
- End of the speed acceleration (point E): identified at the second inflection point of the Doppler radar speed curve^55^. This point marks the beginning of the straight gate phase after the turn.

The first and last turns were excluded from the analyses. To analyse the repeatability over the 3 sessions, the following dependent variables were calculated for the right and left turns between the start of the speed deceleration (A) and the end of the speed acceleration (E):

- Mean of the following time intervals: A to E (AE), A to B (AB), B to C (BC), C to D (CD), B to E (BE), D to E (DE), A to C (AC), A to D (AD), B to D (BD).
- Mean MKF of each step during the turn defined in the AE time interval^56^. The MKFs were identified by peak knee flexion. Steps were defined as a knee flexion followed by a return to the initial value.
- Mean of the MKF of each turn defined in the AE time interval^56^.
- SLL of each turn defined in the AE time interval: $\:Sumload={\sum\:}_{i=1}^{n}\sqrt{\left({x}_{i}^{2}+{y}_{i}^{2}+{z}_{i}^{2}\right)}$ with n the total number of points during a AE turn and i the increment index^45,46^. The SLL was calculated based on the entire AE segment time. We also normalised the SLL by the AE time, since the duration of the turns depends on walking speed.

### Statistical analysis

**Fig. 3:**
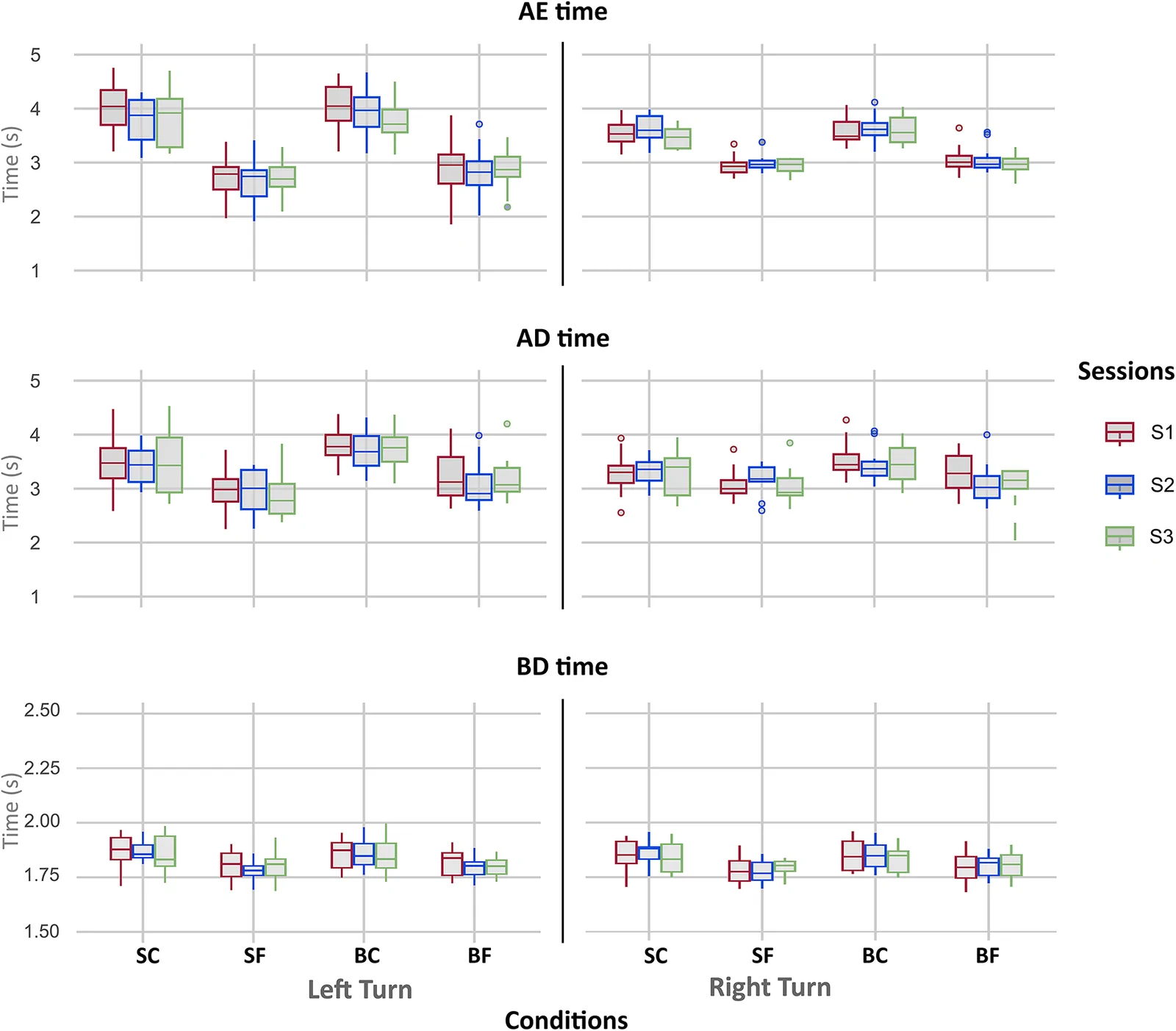
Boxplot describing the time data for the right and left turn phases, depending on the sessions as well as the footwear and speed conditions. The results are presented as the mean of the time. In brackets are the devices needed for recording. AE time, time between the start of the deceleration (before the turn) and the end of the acceleration (after the turn); AD time, time between the start of the deceleration (before the turn) and the end of the body’s rotational turn; BD time, time between the start of the body’s rotational turn and the end of the body’s rotational turn; SC, shoes at comfortable speed; SF, shoes at fast speed; BC, barefoot at comfortable speed; BF, barefoot at fast speed; IMU, inertial measurement unit.

**Fig. 4:**
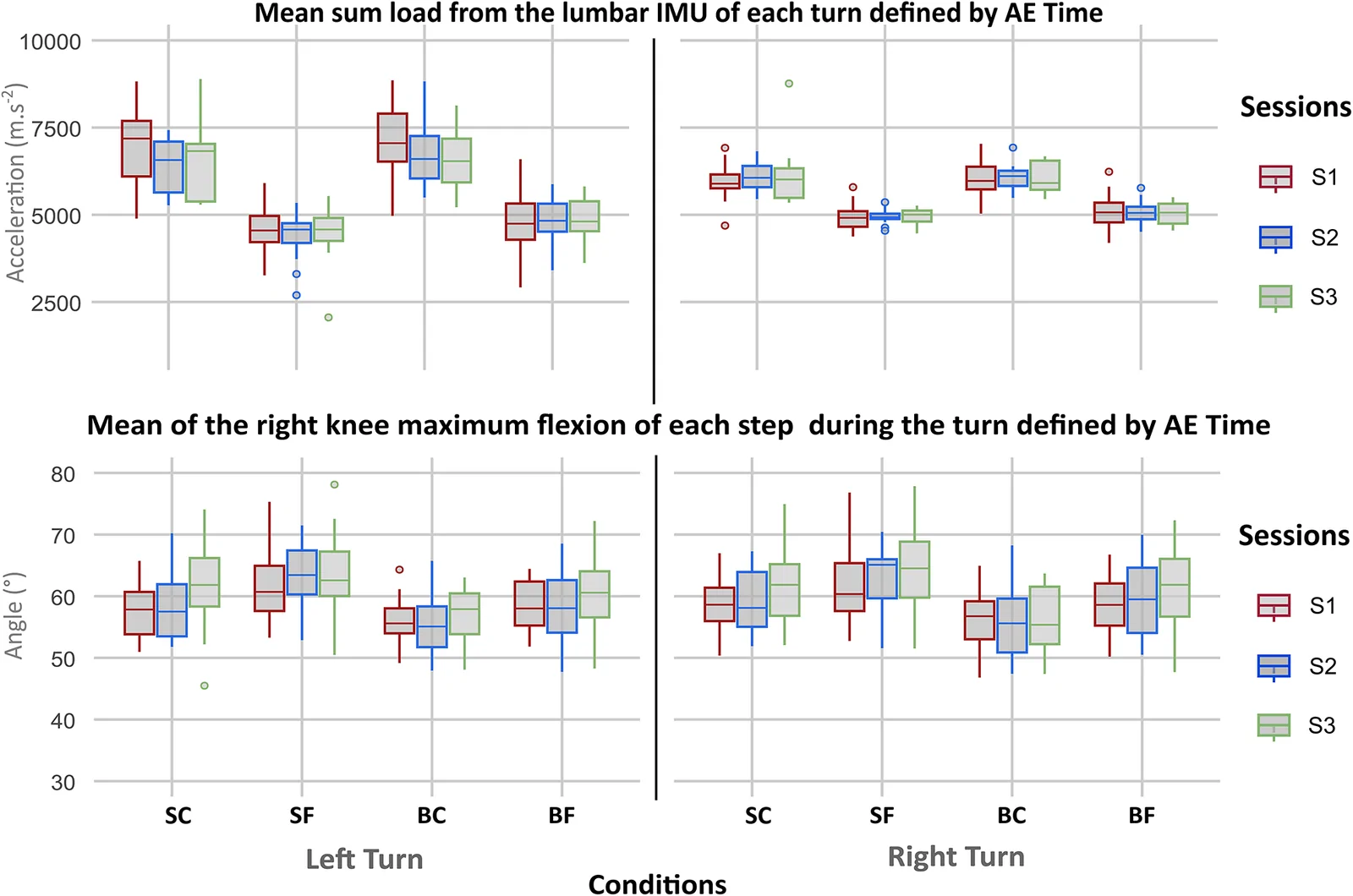
Boxplot describing the gait variables for the right and left turn phases, depending on the sessions as well as the footwear and speed conditions. GRF, ground force reaction; SC, shoes at comfortable speed; SF, shoes at fast speed; BC, barefoot at comfortable speed; BF, barefoot at fast speed.

Statistical analyses were conducted with R (R v R 4.3.2, R Core Team, 2020, R Foundation for Statistical Computing, Vienna, Austria). Descriptive data are presented with boxplots (Figs. 3 and 4). The intersession repeatability agreement was examined for the dependent variables between the 3 sessions and was based on the standard error of measurement (SEM) at 95% confidence level (Eq. 1)^57–59^, the minimal detectable change at 95% confidence level (MDC_95%_) (Eq. 2)^57–59^, and the coefficient of variation (CV) (Eq. 3). The SEM is a measure of reliability that considers multiple sources of variability, such as evaluation and measurement, and expresses repeatability in relation to these sources^57–59^. To guarantee accuracy, the SEM needs to be as low as possible. The objective is for the SEM to be under the standard deviation (SD), which indicates that the error measurement is under the inter-individual variation^47,57^. The MDC_95%_ is the minimum amount of change in a subject’s score that ensures that the change is not due to measurement error, the objective is the MDC_95%_ is to be upper the SD. The objective of the MDC95% is to exceed the SD, indicating that inter-individual variation cannot be considered a difference^60^. The CV provides a clinical estimate of the error between different measurement times. The CV values are presented as percentages, whereas the MDC_95%_ and SEM values have the same units as their respective variables. The mean, standard deviation (SD), CV, SEM, and MDC_95%_ values for the 3 sessions (S1 and S2; S1 and S3; S2 and S3) were averaged to obtain a single value, which is presented in Tables 3 and 4. Variability was considered acceptable if CV value was lower than 10% and unacceptable when the CV value was greater than 20%^61^. We included the two sessions performed in our analysis if a participant did not take part in one of the three sessions, or if sensor noise made processing the data from one of the sessions impossible.

1 $$\:SEM=SD\times\:\sqrt{1-ICC}$$

2 $$\:MDC=SEM\times\:1.96\times\:\sqrt{2}$$

3 $$\:CV=100\times\:\left(\frac{SD/\sqrt{2}}{\mathrm{Mean}\left(Trial\:1,Trial\:2\right)}\right)$$

**Fig. 5:**
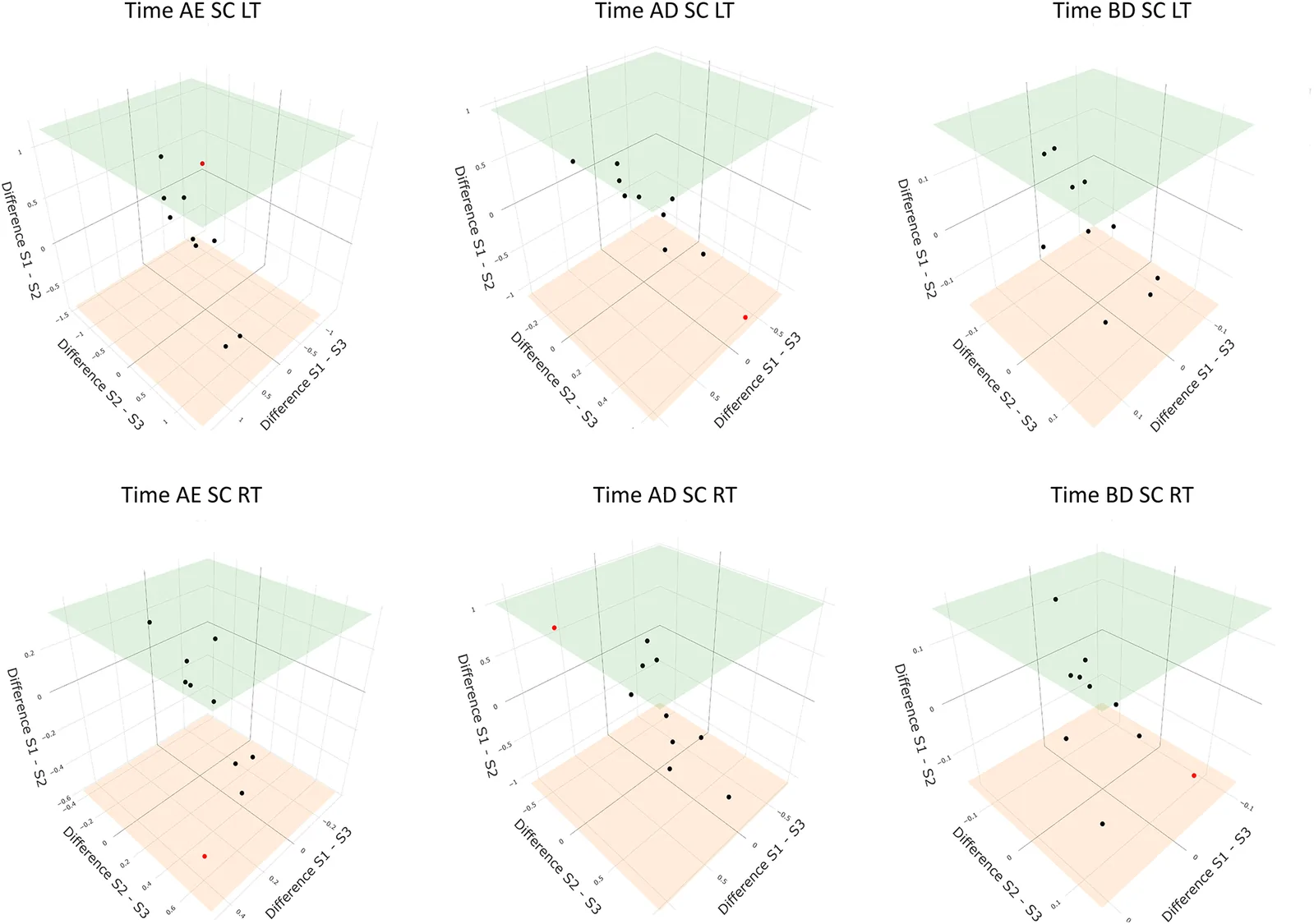
Three-dimensional plots based on the Bland‒Altman method to assess the agreement between the 3 sessions of measurements. In these plots, the values of the differences between the sessions (S1–S2; S1–S3; S2–S3) and their limit of agreement (LOA), upper and lower, are presented on the abscissa and ordinate axes, respectively. The points inside the cube (blue points) represent the participants’ data within the LOA, indicating good repeatability of the measurement. Conversely, if points are outside the cube (red points), the LOA is exceeded but inside 95% confidence interval. x axis is the differences between S1 and S3, Y-axis is the differences between S2 and S3, Z-axis is the differences between S1 and S2, AE time: time between the start of the deceleration (before the turn) and the end of the acceleration (after the turn), AD time: time between the start of the deceleration (before the turn) and the end of the body’s rotational turn, BD time: time between the start of the body’s rotational turn and the end of the body’s rotational turn, SC: shoes at comfortable speed, RT: right turn, LT: left turn. Confidence interval (CI) 95% wasn’t figured on the plot but all the points were inside the maximum LOA CI95%. The dynamic versions are presented in appendix 5, 6, 7, 8, 9 and 10.

Bland‒Altman plot is a common method used for repeatability studies^62^. It plots the difference between paired measurements against their average, revealing bias and agreement. The Bland–Altman plot includes the mean difference and the 95% limits of agreement (LOA; ±1.96 SD). As the data are from three measurement sessions, it was not possible to generate Bland‒Altman plots in 2D with the information from the 3 sessions. We proposed an alternative method by generating 3D plots with the same characteristics as Bland‒Altman plot to assess the agreement between three sets of measurements. In these plots, the values of the differences (S1–S2; S1–S3; S2–S3) and their LOAs are presented in 3D plot in the form of a cube (Fig. 5). The points inside the cube represent the participants’ data within the LOA, indicating good repeatability of the measurement. Conversely, if points are outside the cube, the LOA is exceeded. We also add the common version of Bland-Altman plot in appendix 1. For the Bland–Altman analysis, we only included participants who completed all three sessions, and whose data was not missing due to sensor noise that made processing impossible.

## Results

### Description

Regarding the data that could not be processed due to sensor noise, one participant in BC had data from one session that could not be interpreted. For BF, all participants’ data could be interpreted. Four participants in SC and two participants in SF had data that could not be interpreted for one session.

Figure 3 shows the descriptive results of the phase times for AE, AD and BD across various conditions and sessions, presenting the median and dispersion of the results with the interquartile range. The mean of the sum load from the lumbar IMU, and the mean of the right knee maximum flexion of each step during the turn defined by AE time during the turn are shown in Fig. 4 and it also shows the dispersion of the data. Given the volume of data, detailed descriptions, and results are provided in the supplementary files (Appendix 2 and Appendix 3).

### Repeatability

The results of the repeatability, including mean, SD, CV, SEM, and MDC_95%_, for the time between A and E, A and D, B and D are shown in Table 2, and in appendix 4. The CVs for these measurements consistently remain below 10%, with the mean SEMs of the 3 sessions also remaining below the SD and the mean MDC_95%_ of the three sessions exceeding the SD (Table 2). When comparing sessions 1 and 2, 1 and 3, and 2 and 3, there are some discrepancies between the SEM and the MDC95% and their respective SD values (Appendix 4). Detailed results, including the means, SDs, CVs, and MDC_95%_ values for the time intervals between A and B, A and C, A and D, B and D B and C, C and D, D and E, are provided in Appendix 2. For the time segments B and C, C and D the CVs are less than 10%, for the time segments between A and B, A and C, B and E, the CVs are less than 20%, and for the time segment D and E, Fy and D the CVs exceed 20%. However, for all the time segment mentioned before, the SEMs remain below the SD, and the MDC_95%_ exceeds the SD (Except for the Time segment BC SC LT where the MDC_95%_ is lower than SD), as presented in Appendix 2. The results of the repeatability of the CV, SEM, and MDC_95%_ for the gait variables are shown in Table 3, and in Appendix 4. The CVs for these measurements are consistently less than 10%, with the mean SEMs of 3 sessions remaining below the SD and the mean MDC_95%_ of the 3 sessions above the SD (Table 3). When comparing sessions 1 and 2, 1 and 3, and 2 and 3, there are some discrepancies between the SEM and the MDC95% and their respective SD values (Appendix 4). In addition, for the complementary gait parameters presented in Appendix 3, SEM is always below the SD, and the MDC_95%_ is greater than the SD for all measurements.

**Table 2:** Time description (mean and SD) and repeatability results for the right and left turns, depending on the session as well as the footwear and speed conditions.

Variable	Condition	Laterality	Mean	SD	CV (%)	SEM	MDC_95%_
AE time (s)	BC	RT	3.62	0.25	4.16	0.14	0.40
		LT	3.98	0.47	4.99	0.24	0.65
	BF	RT	3.03	0.22	2.80	0.12	0.34
		LT	2.85	0.44	5.51	0.25	0.70
	SC	RT	3.55	0.23	3.70	0.14	0.39
		LT	3.89	0.48	3.25	0.27	0.74
	SF	RT	2.96	0.15	2.34	0.09	0.24
		LT	2.70	0.37	4.36	0.22	0.61
AD time (s)	BC	RT	3.47	0.33	4.98	0.19	0.52
		LT	3.74	0.37	5.00	0.20	0.56
	BF	RT	3.15	0.34	6.90	0.20	0.55
		LT	3.17	0.42	6.94	0.24	0.66
	SC	RT	3.30	0.35	6.55	0.19	0.52
		LT	3.56	0.62	4.81	0.25	0.69
	SF	RT	3.08	0.31	5.80	0.18	0.50
		LT	2.92	0.41	4.45	0.24	0.66
BD time (s)	BC	RT	1.84	0.06	1.35	0.04	0.10
		LT	1.85	0.07	1.99	0.04	0.65
	BF	RT	1.80	0.06	0.96	0.03	0.34
		LT	1.80	0.05	1.42	0.03	0.70
	SC	RT	1.85	0.06	1.18	0.04	0.10
		LT	1.86	0.08	2.61	0.04	0.12
	SF	RT	1.78	0.05	1.73	0.03	0.08
		LT	1.80	0.07	2.52	0.04	0.11

AE time, time between the start of the deceleration (before the turn) and the end of the acceleration (after the turn); AD time, time between the start of the deceleration (before the turn) and the end of the body’s rotational turn; BD time, time between the start of the body’s rotational turn and the end of the body’s rotational turn; SC, shoes at comfortable speed; SF, shoes at fast speed; BC, barefoot at comfortable speed; BF, barefoot at fast speed; RT, right turn; LT, left turn; SD, standard deviation; CV, coefficient of variation; SEM, standard error of measurement; MDC_95%_, minimal detectable change at the 95% confidence level.

**Table 3:** Gait parameter descriptions (means and SDs) and repeatability results for the right and left turns, depending on the session as well as the footwear and speed conditions.

Variable	Condition	Laterality	Mean	SD	CV (%)	SEM	MDC_95%_
Mean sum load from the lumbar IMU of each turn defined by AE Time (m s^− 2^)	BC	RT	6047.60	471.83	4.57	274.63	761.25
		LT	6807.58	970.08	6.95	524.96	1455.12
	BF	RT	5071.02	406.45	3.30	233.33	646.76
		LT	4812.29	773.35	6.69	441.48	1223.73
	SC	RT	6049.50	632.09	2.93	282.48	783.00
		LT	6653.28	1005.88	3.77	602.15	1669.06
	SF	RT	4948.81	289.07	3.09	175.32	485.97
		LT	4452.00	773.24	8.83	460.87	1277.47
Mean sum load from the lumbar IMU normalised by AE time (m s^− 3^)	BC	RT	1672.59	64.97	3.32	39.52	109.53
		LT	1675.98	60.33	3.03	36.29	100.59
	BF	RT	1676.60	72.37	3.61	43.34	120.14
		LT	1686.71	74.95	3.86	44.68	123.85
	SC	RT	1679.54	61.58	3.37	39.05	108.23
		LT	1688.26	60.47	3.03	37.82	104.83
	SF	RT	1673.39	61.03	3.08	36.48	101.13
		LT	1680.66	66.37	3.67	40.95	113.52
Mean of the right knee maximum flexion of each step during the turn defined by AE Time	BC	RT	56.23	5.33	5.66	3.08	8.54
		LT	56.09	4.51	4.96	2.64	7.31
	BF	RT	59.69	6.19	6.22	3.59	9.95
		LT	59.00	6.03	6.23	3.52	9.77
	SC	RT	59.92	5.64	5.21	3.34	9.25
		LT	59.18	5.92	8.14	3.58	9.93
	SF	RT	63.07	6.60	3.54	3.96	10.99
		LT	62.97	6.22	5.20	3.73	10.34

AE time, time between the start of the deceleration (before the turn) and the end of the acceleration (after the turn); SC, shoes at comfortable speed; SF, shoes at fast speed; BC, barefoot at comfortable speed; BF, barefoot at fast speed; RT, right turn; LT, left turn; SD, standard deviation; CV, coefficient of variation; SEM, standard error of measurement; MDC_95%_, minimal detectable change at the 95% confidence level.

### 3D plot based on the Bland‒Altman method

In condition SC, AE, and AD modalities have only 1 point for RT and LT outside the LOA, and for BD, only one point is in RT. All the points were inside the maximum LOA confidence interval of 95% (Fig. 5).

## Discussion

The aim of this study was to test the repeatability of three different sessions of a new, dynamic, and nonintrusive method to analyse curved gaits at spontaneous walking speeds, at fast walking speeds, and both barefoot and with shoes. This is the first study to examine the repeatability of a gait assessment protocol during an instrumented F8W of 9 m involving 1.2 m curved bends, with a particular focus on the different phases of the turn, i.e., speed deceleration, body rotation, and speed acceleration.

Figures 3 and 4 show the consistency of our measurements by presenting values that align with the requested speeds for the three sessions. However, due to their descriptive nature, it is not possible to draw statistical conclusions from them.

The main findings of our study are the repeatability across all conditions (BC, BF, SC, SF) of AE time, AD time and BD time for left and right laterality. For each condition and laterality, the CV, mean SEM, and mean MDC_95%_ attest to repeatability. Figure 5, which is based on Bland–Altman, shows the repeatability of the segmentation of the turn with SC and tends to show the repeatability of the turn of the participants during 3 sessions. The AD time is of great interest. It is the time between the start of deceleration A and the end of the body’s rotational turn D, with A being measured using Doppler radar and D using an IMU. Using two different devices to measure AD demonstrates the consistency of our protocol and reinforces the procedure of external synchronisation.

The results for the AB time, AC time, and BE time were more mixed, with a CV between 10% and 20% but still a SEM lower than the SD and an MDC_95%_ higher than the SD. The DE and FyD times were found to be non-repeatable. We can consider the fact that two devices are always needed to determine them: Doppler radar with a lumbar IMU (AB, AC, BE, AC, DE). These measures are repeatable with a CV of less than 20% except for DE. Since these results were produced for an F8Wi of 9 m and 1.2 m curved bends, they cannot be currently extrapolated to the traditional figure of 8 of 1.5 m^13^.

The remaining dependent gait variables were measured between time points A and E. These included mean SLL of each turn, mean sum load from the lumbar IMU normalised by AE time, mean of the right knee maximum flexion of each step during the turn. These variables were found to be repeatable. These findings emphasise the stability of the measurements within this new definition of a turn during an F8Wi. Exploring gait parameters and their differences in each laterality within this new protocol could be a research objective.

We found that the interval between the commencement of deceleration (prior to body rotation) and the conclusion of acceleration (after body rotation) (AE time) is considerably longer than the time interval solely determined by body rotation (BD time). The time determined by the vertical rotation is consistent with the results of the study on the assessment of the TUG with the IMU^63^. The mean duration of the 180° rotation during the time up and go test in the group of young subjects was approximately 1.85 ± 0.16 s^63^. In our study, the time between B and D, at a comfortable speed with shoes, was on average 1.82 ± 0.06 s. Another study found that, using motion capture and a cut-off of 30° s^− 2^ for the axial pelvic rate, participants took 2.23 ± 0.7 s to perform the turn at spontaneous speed^64^. However, we observed that the time between the reduction in speed and the increase in speed (AE) in this condition was 3.7 ± 0.4 s. This time shows that the change in speed and its association with the turn occurred before the start of the pelvic rotation. So, to get an overview of the turn, we can’t limit the segmentation of the turn to the rotation of the pelvis. In addition, speed variation is a key element of the turn, so the distance needed to reach a comfortable speed should be considered. The distance of the traditional F8W is 1.50 m^13,14^, yet the distance for older people to reach a steady walking speed during straight walking exceeds 1.46 m^65^. This means that the distance of the traditional F8W may be insufficient or nonphysiological for elderly people and other specific populations. Moreover, the deceleration zone influences the straight walking speed^23^, and multiple mechanisms of compensation occur during acceleration and deceleration^19^. These two phases seem to be capital for overlooking the turning phase. König et al.^66^ chose a distance of 11.52 m for their protocol, which was long enough to ensure that enough gait cycles were needed for reliable motion capture. However, the main limitation of this repeatable study by König et al.^66^ was that it analysed only straight walking segments of the F8W.

Our results seem to show that some gait parameters and turning points of interest are repeatable during a turn. Future studies should investigate how these gait parameters vary during turning. One of the main questions that arises is as follows: are the right and left turns symmetrical? Strike et al.^67^ concluded that some gait parameters are asymmetrical, but the results and the repeatability of the measurements allow us to emphasise that the right and left turns remained stable throughout the 3 sessions.

The F8W has been described in numerous studies^13–15,17,18,25^ but with shoes at a comfortable speed only. The present experiment shows that our protocol of the F8Wi is repeatable through different conditions. While Hollander et al.^38^ reported differences in gait parameters when walking barefoot or in shoes in a straight line, future studies should explore differences in gait conditions when turning, and how this influences turning strategies in terms of speed variation and body rotation.

### Limitations

Despite the fact the mean SEM and mean MDC_95%_ shown elements of repeatability, there are some concerns about the comparison of SEM and MDC 95% when comparing sessions 1 and 2, 1 and 3, and 2 and 3. It cannot be explained by learning alone because there are SEM upper and lower SDs for the comparison between sessions 2 and 3. This inconsistency can be attributed to the natural variation in individuals from one day to the next. In effect, there is greater variation in healthy individuals between sessions for standard walking conditions than for conditions requiring cognitive function, which is the inverse for people with multiple sclerosis^68^. As gait during turning is presented with low cognitive requirements and without a dual task for healthy participants, this study with frontal near infrared spectroscopy^4^ found no significant difference in prefrontal cortex activation between standard waking and waking during an F8W. These elements of variation F8Wi would need to be explored for clinical studies.

Our study used a distance of 9.10 m between the two turns, which may have given the participants the opportunity to reach a steady walking speed between the turns and break their speed as they approached the turns. However, one of the main limitations of this distance is its applicability to clinical evaluation. In the case of a stroke, for example, the distance walked can be less than that of healthy participants^69^, as participants who need a four-point cane to walk only reach 101 m^70^. Therefore, the distance presented in our study appears excessive due to the repetition of the 4 shape of 8, which is at least 72.8 m. It would be interesting to compare the strategies of speed variation and body rotation in the traditional figure of eight and in a figure of eight with a longer distance between the turns, and to consider how these elements would be affected by pathologies.

The devices used in this study, i.e., Doppler radar, IMU, electrogoniometers, but only two devices are necessary for measuring the time AE, BD and AD: Doppler radar and IMU. By limiting the number of devices, this protocol would become more accessible to different teams exploring curved walking in research and in clinical practice. An idea would be to exclude the radar to evaluate speed. The speed acceleration would be derived from the IMU to calculate the speed^71^, and the IMU would only be used for segmenting the different turn phases. However, at the moment, the IMU protocol for evaluating speed during turns does not provide the participant’s instantaneous speed due to the IMU’s technical problem with derivation over time^72^. Instead, it calculates the average speed of the participant during the testing procedure or the average stride speed by counting the number of stride with the IMU normalised by the participant’s height^73^, or it may use the invert pendulum to calculate the speed during the turn^72^. At present, these elements have only been explored in healthy participants. Further studies on the validity of this method are needed before it can be used in clinical studies.

In addition, the speed evaluation by Doppler radar raised some concerns due to the difference in the distribution profile between the right and left turns, as shown in Fig. 3. The Doppler radar was located close to the right turn, which could result in differences between the data collected from the left and right turns. As Zhang et al.^74^, describe, analysing the curved phase can lead to problems relating to equipment and data handling, which do not occur when analysing a straight line. Using two synchronised radars to explore each side of the turn could reinforce our measurements, but this approach would still be restrictive. Nevertheless, the results of AE and AD are repeatable, as presented above.

## Conclusion

This study demonstrated that the time of specific turn phases (e.g., AE time, AD time, BD time) and certain gait parameters during turn defined by AE time (e.g. mean SLL of each turn, mean sum load from the lumbar IMU normalised by AE time, mean of the right knee maximum flexion of each step during the turn) are repeatable for this modified F8W. Despite existing discrepancies, the measurements are repeatable for both right and left turns, and for all conditions, including those involving the use of shoes at a comfortable and fast speed, as well as those conducted barefoot at a comfortable and fast speed. Further studies are needed to explore the differences in terms of strategy when it comes to time segmentation for right and left turns. Clinical studies are needed to explore the applicability of this new measurement to gait evaluation during turns in people with pathologies (e.g. neurological and orthopaedic disorders).

## Supplementary Information

Below is the link to the electronic supplementary material.

Supplementary Material 5

Supplementary Material 6

Supplementary Material 7

Supplementary Material 8

Supplementary Material 9

Supplementary Material 10

Supplementary Material 4

Supplementary Material 1, 2, 3

## Data Availability

For data requests, contact the corresponding author.
