# Supplementary material for: Exploring the repeatability of curved gait during an instrumented figure of 8 walk test, a new methodology
Source: Sci Rep. 2026 Jun 18;16:27979. doi: 10.1038/s41598-026-57567-2 (PMC13550482; doi:10.1038/s41598-026-57567-2)
Supplement: Supplementary file 1 [file 41598_2026_57567_MOESM8_ESM.docx]

# Supplementary

*Appendix 1.* Bland–Altman plot assessing the agreement across three measurement sessions for the condition “shoes at comfortable speed (CS)”. The plot shows AE time (the time between the onset of deceleration before the turn and the end of acceleration after the turn), AD time (the time between the onset of deceleration before the turn and the end of the body’s rotational turn), and BD time (the time between the onset and the end of the body’s rotational turn). Abbreviations: RT, right turn; LT, left turn; LOA, limits of agreement; CI, confidence interval.

*appendix 2. Description (mean and SD) and repeatability results for the right and left turns, depending on the session as well as the footwear and speed conditions.*

| **variable** | **Condition** | **Laterality** | **Mean** | **SD** | **CV (%)** | **SEM** | **MDC_95%_** |
| --- | --- | --- | --- | --- | --- | --- | --- |
| AB | BC | RT | 1.63 | 0.32 | 9.98 | 0.18 | 0.50 |
|  |  | LT | 1.89 | 0.39 | 8.32 | 0.21 | 0.51 |
|  | BF | RT | 1.36 | 0.28 | 15.26 | 0.16 | 0.43 |
|  |  | LT | 1.26 | 0.29 | 14.93 | 0.17 | 0.60 |
|  | SC | RT | 1.46 | 0.34 | 18.59 | 0.18 | 0.51 |
|  |  | LT | 1.75 | 0.40 | 8.53 | 0.18 | 0.59 |
|  | SF | RT | 1.24 | 0.26 | 16.67 | 0.16 | 0.45 |
|  |  | LT | 1.13 | 0.37 | 15.23 | 0.22 | 0.48 |
| BC | BC | RT | 0.92 | 0.04 | 3.17 | 0.02 | 0.06 |
|  |  | LT | 0.93 | 0.06 | 5.02 | 0.03 | 0.09 |
|  | BF | RT | 0.90 | 0.04 | 3.46 | 0.02 | 0.06 |
|  |  | LT | 0.91 | 0.11 | 6.61 | 0.06 | 0.17 |
|  | SC | RT | 0.92 | 0.04 | 3.40 | 0.03 | 0.07 |
|  |  | LT | 0.94 | 0.11 | 5.50 | 0.03 | 0.08 |
|  | SF | RT | 0.88 | 0.03 | 3.33 | 0.02 | 0.05 |
|  |  | LT | 0.89 | 0.04 | 3.68 | 0.02 | 0.07 |
| CD | BC | RT | 0.92 | 0.04 | 2.53 | 0.02 | 0.06 |
|  |  | LT | 0.94 | 0.09 | 5.60 | 0.05 | 0.14 |
|  | BF | RT | 0.90 | 0.04 | 2.47 | 0.02 | 0.06 |
|  |  | LT | 0.93 | 0.12 | 6.73 | 0.07 | 0.20 |
|  | SC | RT | 0.92 | 0.04 | 2.52 | 0.02 | 0.06 |
|  |  | LT | 0.94 | 0.07 | 4.64 | 0.03 | 0.08 |
|  | SF | RT | 0.90 | 0.03 | 2.58 | 0.02 | 0.05 |
|  |  | LT | 0.90 | 0.04 | 3.51 | 0.02 | 0.06 |
| DE | BC | RT | 0.38 | 0.28 | 47.22 | 0.16 | 0.45 |
|  |  | LT | 0.43 | 0.41 | 76.98 | 0.24 | 0.66 |
|  | BF | RT | 0.41 | 0.54 | 64.23 | 0.31 | 0.87 |
|  |  | LT | 0.54 | 0.56 | 67.93 | 0.32 | 0.90 |
|  | SC | RT | 0.51 | 0.54 | 58.93 | 0.31 | 0.87 |
|  |  | LT | 0.66 | 0.65 | 61.71 | 0.39 | 1.07 |
|  | SF | RT | 0.28 | 0.23 | 64.79 | 0.14 | 0.38 |
|  |  | LT | 0.37 | 0.31 | 66.53 | 0.19 | 0.52 |
| AC | BC | RT | 2.54 | 0.31 | 7.68 | 0.17 | 0.48 |
|  |  | LT | 2. 86 | 0.43 | 6.67 | 0.23 | 0.64 |
|  | BF | RT | 2.25 | 0.30 | 10.59 | 0.19 | 0.52 |
|  |  | LT | 2.20 | 0.36 | 11.74 | 0.22 | 0.61 |
|  | SC | RT | 2.35 | 0.41 | 12.86 | 0.23 | 0.64 |
|  |  | LT | 2.68 | 0.65 | 9.91 | 0.28 | 0.78 |
|  | SF | RT | 2.18 | 0.33 | 7.29 | 0.19 | 0.54 |
|  |  | LT | 1.98 | 0.41 | 8.47 | 0.24 | 0.67 |
| BE | BC | RT | 2.01 | 0.40 | 14.26 | 0.23 | 0.63 |
|  |  | LT | 2.18 | 0.35 | 11.77 | 0.20 | 0.54 |
|  | BF | RT | 1.74 | 0.42 | 12.14 | 0.25 | 0.69 |
|  |  | LT | 1.73 | 0.43 | 12.60 | 0.25 | 0.69 |
|  | SC | RT | 2.16 | 0.52 | 10.16 | 0.28 | 0.77 |
|  |  | LT | 2.24 | 0.65 | 12.40 | 0.36 | 1.00 |
|  | SF | RT | 1.70 | 0.36 | 12.96 | 0.21 | 0.59 |
|  |  | LT | 1.55 | 0.42 | 18.03 | 0.25 | 0.70 |

*Abbreviations: AB time: time between the start of the deceleration (before the turn) and the start of the body’s rotational turn; BC time: time between the start of the body’s rotational turn and* *the maximum of the body’s rotational turn; CD time: time between the maximum of the body’s rotational turn and time between the end of the body’s rotational turn; AC time: time between the start of the deceleration (before the turn) and the maximum of the body’s rotational turn; BE time: time between the start of the body’s rotational turn and the end of the acceleration (after the turn); DE time : time between* *the end of the body’s rotational turn and the end of the acceleration (after the turn); SC: shoes at comfortable speed; SF: shoes at fast speed; BC: barefoot at comfortable speed; BF: barefoot at fast speed; RT: right turn; LT: left turn; SD: standard deviation; CV: coefficient of variation; SEM: standard error of measurement; MDC_95%_: minimal detectable change at the 95% confidence level.*

**Appendix 3.** Description (mean and SD) and repeatability results for the right and left turns, depending on the session as well as the footwear and speed conditions.

| Variable | Condition | Laterality | Mean | SD | CV (%) | SEM | MDC95% |
| --- | --- | --- | --- | --- | --- | --- | --- |
| Mean of the left knee maximum flexion of each step during the turn defined by AE Time (°) | BC | RT | 58.21 | 6.07 | 6.94 | 3.7 | 10.3 |
|  |  | LT | 57.63 | 6.64 | 7.07 | 4.0 | 11.0 |
|  | BF | RT | 60.30 | 6.08 | 6.37 | 4.1 | 11.5 |
|  |  | LT | 59.70 | 6.14 | 6.23 | 4.2 | 11.6 |
|  | SC | RT | 60.67 | 6.42 | 5.72 | 3.6 | 10.1 |
|  |  | LT | 60.48 | 6.82 | 6.97 | 4.2 | 11.5 |
|  | SF | RT | 63.33 | 6.38 | 6.61 | 4.3 | 11.8 |
|  |  | LT | 62.80 | 7.41 | 7.16 | 5.0 | 13.9 |
| Mean of the right knee maximum flexion of each step during the turn defined by AE Time (°) | BC | RT | 56.23 | 5.33 | 5.66 | 3.08 | 8.54 |
|  |  | LT | 56.09 | 4.51 | 4.96 | 2.64 | 7.31 |
|  | BF | RT | 59.69 | 6.19 | 6.22 | 3.59 | 9.95 |
|  |  | LT | 59.00 | 6.03 | 6.23 | 3.52 | 9.77 |
|  | SC | RT | 59.92 | 5.64 | 5.21 | 3.34 | 9.25 |
|  |  | LT | 59.18 | 5.92 | 8.14 | 3.58 | 9.93 |
|  | SF | RT | 63.07 | 6.60 | 3.54 | 3.96 | 10.99 |
|  |  | LT | 62.97 | 6.22 | 5.20 | 3.73 | 10.34 |
| Mean of the left knee maximum flexion of each turn defined by AE Time (°) | BC | RT | 61.61 | 6.35 | 6.70 | 3.70 | 10.26 |
|  |  | LT | 60.29 | 6.83 | 6.44 | 3.98 | 11.03 |
|  | BF | RT | 66.03 | 7.24 | 7.07 | 4.13 | 11.46 |
|  |  | LT | 64.23 | 7.28 | 6.15 | 4.18 | 11.59 |
|  | SC | RT | 64.45 | 6.60 | 5.71 | 3.64 | 10.08 |
|  |  | LT | 63.95 | 7.28 | 8.03 | 4.16 | 11.54 |
|  | SF | RT | 70.31 | 7.06 | 7.13 | 4.26 | 11.80 |
|  |  | LT | 67.92 | 8.37 | 7.81 | 5.02 | 13.91 |
| Mean of the right knee maximum flexion of each turn defined by AE Time (°) | BC | RT | 59.70 | 5.90 | 4.59 | 3.44 | 9.53 |
|  |  | LT | 59.01 | 4.79 | 6.97 | 2.79 | 7.73 |
|  | BF | RT | 65.69 | 7.74 | 6.96 | 4.43 | 12.27 |
|  |  | LT | 63.30 | 6.97 | 7.20 | 4.06 | 11.25 |
|  | SC | RT | 63.53 | 6.44 | 6.23 | 3.95 | 10.96 |
|  |  | LT | 62.36 | 6.65 | 9.94 | 4.16 | 11.53 |
|  | SF | RT | 69.94 | 7.62 | 3.43 | 4.57 | 12.68 |
|  |  | LT | 68.18 | 6.81 | 5.94 | 4.08 | 11.30 |

*Abbreviations: SC: shoes at comfortable speed, SF: shoes at fast speed, BC: barefoot at comfortable speed, BF: barefoot at fast speed, RT: right turn, LT: left turn, SD: standard deviation, CV: coefficient of variation, SEM: standard error of measurement, MDC_95%_: minimal detectable change at the 95% confidence level.*
